# Efficacy and safety of Chinese herbal medicine Long Dan Xie Gan Tang in insomnia: A systematic review and meta-analysis

**DOI:** 10.1097/MD.0000000000019410

**Published:** 2020-03-13

**Authors:** Xu Fan, Zhuang Su, Shichao Nie, Jie Yang, Xiaoyu Zhang, Dongyang Tan, Sihang Xie, Yi Xu, Yanying Zhao, Liu Feng, Mingyi Gu, Xiaomeng Sun

**Affiliations:** Liaoning University of Traditional Chinese Medicine, Shenyang, Liaoning province, China.

**Keywords:** insomnia, Long Dan Xie Gan Tang, meta-analysis, randomized control trial

## Abstract

**Background:**

Insomnia is a global public problem, which has a significant negative impact on both physical and mental health, while increasing the economic burden placed on both sufferers and society. Western medicine has a fast treatment on sleep, but it leads to side effects and strong dependence. Long Dan Xie Gan Tang(LDXGT) is a representative Chinese herbal medicine for the treatment of insomnia especially which has a bad-tempered symptom, and its effectiveness and safety has been validated clinically. However, there is yet to be any evidence-based medicine. Therefore, the effectiveness and safety of LDXGT in the treatment of insomnia are studied and systematically evaluated in this study. It will provide a theoretical support for the treatment of insomnia compared to western medicine.

**Objective:**

The study is purposed to evaluate the effectiveness and safety of LDXGT for the treatment of insomnia.

**Methods:**

Search was conducted for various databases including Pubmed, Chinese Biomedicine Database(CBM), China National Knowledge Infrastructure (CNKI), Chinese Scientific Journals Database (VIP), and Wan-fang. Randomized-controlled trials (RCTs) were identified for insomnia treatment involving LDXGT and LDXGT combined with ordinary Western medicine. The quality of literature was evaluated by Cochrane assessing tool to reduce the risk of bias. RevMan 5.3 software and STATA 12.0 software were applied to perform the meta-analysis.

**Results:**

Thirteen studies involving 1181 participants were identified in this systematic review. Few studies described the details of random principle. No placebo was involved in treatment. LDXGT was compared with ordinary Western medicine in 11 trials and with LDXGT combined with conventional Western medicine in 2 trials. The results of our meta-analysis showed the relative benefits in effective rates compared with conventional western medicine. (Odds Ratio [OR]= 4.32, *I*^2^ = 0%,95% confidence interval CI [3.05 to 6.13], *P* < .00001) and recovery rate was (Odds Ratio [OR] = 2.67, *I*^2^ = 0%,95% confidence interval CI [2.04 to 3.48], *P* < .00001). In two trials, adverse events were reported, but no serious adverse effects were reported.

**Conclusion:**

Our systematic evaluation will provide evidence for the clinical effectiveness and safety of LDXGT in the treatment of insomnia, and the side effects of western medicine are addressed. Further trials are necessary to collect the evidence for the use of LDXGT.

## 1 Introduction

Insomnia usually refers to a subjective experience in which patients are not satisfied with their sleep time or quality as their daytime social activities are affected, and it is mainly manifested in the difficulty in falling asleep, sleep maintenance disorder, early awakening, decline in sleep quality, and decrease in total sleep time. It is usually accompanied by daytime dysfunction.^[1]^ Insomnia as a global public health problem, could have a significant negative impact on both physical and mental health, while increasing the economic burden placed on both sufferers and society. The existing clinical therapeutic methods mainly include benzodiazepine receptor agonists. The second-generation benzodiazepines are widely used in the treatment of insomnia, as they can reduce the time taken to fall asleep, reduce the awakening time and increase the duration of sleep.^[2]^ However, this sort of medicine also produces side effects and causes negative emotions. It tends to result in drug dependence and even beyond the therapeutic effect, the patient is intolerable. Therefore, this study is purposed to provide a new method for the treatment of insomnia.

LDXGT is the formula that can remove heat from the organs by TCM.^[17]^ It originates from the Formulae of the Bureau of People's Welfare Pharmacies (Taiping Huimin Hejiju Fang in Chinese) in the 960s to 1127 s. The LDXGT decoction is composed of 10 Chinese herbal medicine, including Chaihu(Radix Bupleuri), Huangqin(Scutellaria Baicalensis), Longdancao(Radix Gentianae), Zhizi(Gardenia Jasminoides Ellis), ZeXie(Rhizoma Alismatis), Mutong(Caulis Akebiae), Cheqianzi(Semen Plantaginis), Danggui(Radix Angelicae Sinensis), Shengdi(Radix Rehmanniae Recen), Gancao(Radix Glycyrrhizae). All types of herbs combined play a role in their effectiveness. Duo to its considerable effect and low cost, LDXGT has been widely applied. The treatment of insomnia with LDXGT is commonly seen in China and it provides a strong theoretical support by TCM. Not only can it improve the quality of sleep, it also dispels the negative emotions caused by insomnia, such as bad temper. In addition, the effect of LDXGT in the treatment of insomnia has yet to be evaluated in the previous system reviews.

## 2 Methods

### 2.1 Eligibility criteria

#### 2.1.1 Types of studies design

Randomized controlled trials (RCTs) on Long Dan Xie Gan decoction in the treatment of insomnia published in China were enrolled. Manuscript published in Chinese and English were permitted. There is no relevant report abroad.

#### 2.1.2 Types of participants

These RCTs comply with the diagnostic criteria of insomnia commonly applied clinically (Chinese medicine diagnostic criteria or Western diagnostic criteria are not limited), with secondary insomnia excluded. The extracted studies are all published raw materials. Participants were recruited regardless of gender, age, and ethnic group.

#### 2.1.3 Types of interventions

The experimental group received single therapy with the LDXGT alone or in combination with western medicine and TCM, regardless of dosage and duration of the treatment. The control group received single therapy involving western medicine.

#### 2.1.4 Inclusion criteria

①all the included literature was in accordance with the clinical RCT trial, using blind method, both Chinese and English literature. ②all the included studies met the diagnostic criteria for insomnia. ③the literature has sufficient data to support the evaluation.

#### 2.1.5 Exclusion criteria

①non-clinical RCT trial and fundamental research. ②does not meet the diagnostic criteria of primary insomnia. ③with serious diseases of internal organs. ④long-term use of other related drugs intended to treat insomnia. ⑤clinical review and case report.

#### 2.1.6 Elimination criteria

We review the included literature thoroughly. If it meets any of the following criteria, it will be eliminated: ① any of the documents in compliance with the exclusion criteria. ②included the incomplete literature data reports. ③identical content of literature. ④combined with other diseases.

#### 2.1.7 Types of outcome measures

We choose the effective rate and the cure rate as the outcome measures. According to the guidance provided by the Clinical Research of New Chinese Medicine,^[3]^ the evaluation criteria for clinical therapeutic effects are as follows: ①clinical cure: nocturnal sleep time >6 hours, good sleep quality, full of energy after waking up. ②noticeably effective: the sleep time at night increased by more than 3 hours. ③Effective: the increase of sleep time at night was less than 3 hours. ④ineffective: Insomnia symptoms were insignificantly improved compared with those before treatment. Cure rate=Clinical/Total number of cases. The total effective rate = (Clinical cure+ Markedly effective + Effective)/Total number of cases × 100%.

### 2.2 Search methods for the identification of studies

#### 2.2.1 Electronic searches

The network electronic databases were searched online. We retrieved Pubmed, Chinese Biomedicine Database(CBM), China National Knowledge Infrastructure (CNKI), Chinese Scientific Journals Database (VIP), and Wan-fang Data. A combination of keywords and free words were performed as the retrieval strategy.

#### 2.2.2 Search strategy

The literature is sourced from PubMed, Chinese Biomedicine Database (CBM), China National Knowledge Infrastructure (CNKI), Chinese Scientific Journals Database (VIP), and WanFang Datebase. Different search strategies were combined as follows: for PubMed, the English search query included: (“Long Dan Xie Gan Tang”[Title/Abstract], “Long Dan Xie Gan Detection”[Title/Abstract]) AND (“insomnia”[Mesh terms], “hyposomnia”[Mesh terms],“sleeplessness”[Mesh terms], “sleep disorders”[Mesh terms]);for the 4 Chinese database, the search query included: “Long Dan Xie Gan Tang” AND “Shi Mian”,“Bu Mei”,“Bu De Mian”. The retrieval time spans from the construction of the database to May 1, 2019. No language limitations were imposed.

### 2.3 Data extraction and analysis

#### 2.3.1 Data extraction and quality assessment

Two evaluators read the title, abstract and full text independently, screen the research and extract the data according to the above inclusion criteria and exclusion criteria. If there is any discrepancy, a third researcher will assist in solving the problem. The evaluators assessed the included literature according to the risk criteria of Cochrane collaborative network bias evaluation. The main evaluation criteria were random distribution method, allocation scheme concealment, blind method, end data integrity, selective report research results, and so on. Finally, the outcome indexes of each clinical study, including therapeutic effect index and safety index, were collected.

#### 2.3.2 Statistical analysis

According to the RevMan 5.3 software provided by Cochrane collaboration network, the statistic data were represented by odds risk (OR), 95% confidence interval (CI), and *I*^2^. The size of the heterogeneity across trials was evaluated using the *I*^2^ statistics. When *P* > .1, *I*^2^ < 50%, suggesting that the heterogeneity between the studies was little, and the fixed-effect merging model was applied. When *P* < .1, *I*^2^ > 50%, the heterogeneity between the studies was significant, and the random-effect merging model was adopted. A single study that could not be combined was analyzed separately, and the clinical outcome was reported according to the description made in the study. After excluding the causes of clinical heterogeneity, random-effect mode analysis and forest map should be used, and the fixed-effect model should be applied to detect the differences between groups. Funnel diagrams should be drawn for publication bias testing.

### 2.4 Ethical approval

This study did not require ethical approval or informed consent of patients, because we just collected the published data from research papers. And the system review itself did not involve patient recruitment. The ethical approval and the consent of patients were not necessary.

## 3 Results

### 3.1 Literature retrieval results

**Figure 1:**
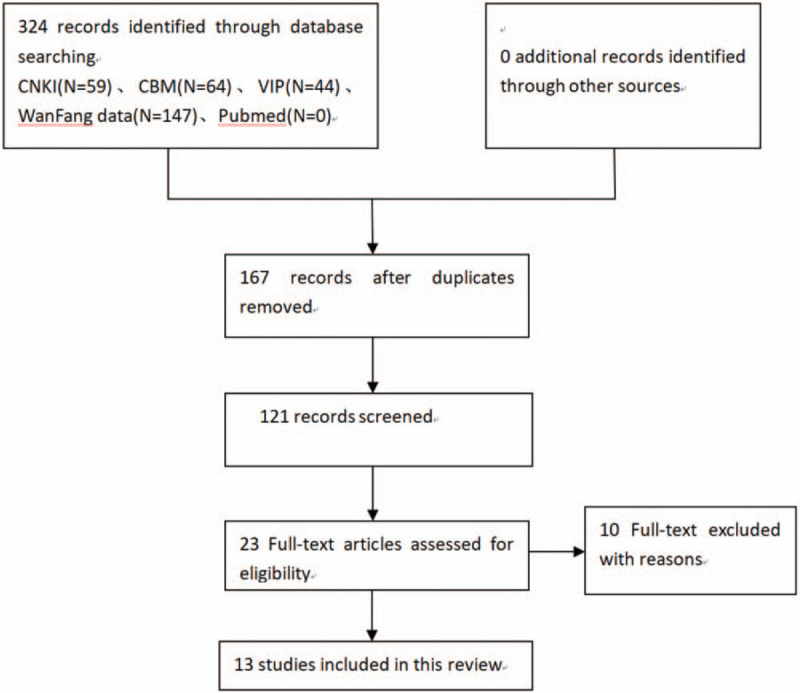
Flow chart of study selection. The flow diagram indicates the retrieval process of the study. The Initial search identified 324 relevant articles, and 121 articles were obtained after duplicates were deleted. After screening the full texts carefully,13 trials were included in this meta-analysis; Flow chart of study selection.

Three hundred twenty four articles were collected, all of which were published studies. We excluded the animal experiments, non-randomized controlled trials, case reports, reviews, theories, and expert experience. Totally 121 studies were obtained after duplicate records were deleted, and another 98 articles were removed. Therefore, 23 full-text articles were assessed for eligibility. After a detailed assessment of the remaining papers, an additional 10 articles were excluded mainly because they failed to satisfy our predetermined inclusion criteria. Finally, 13 RCTs (1181 patients) were collected, including 573 patients in the experimental group and 608 patients in the control group (Fig. 1). The duration of treatment varied from 2 weeks to 4 weeks. Adverse effects were reported in 2 studies in the control group, while there was no mention made in the other studies.

### 3.2 General characteristics of literature

The treatment group included in the study used LDXGT as the related treatment, 11 articles were treated with LDXGT alone compared with western medicine, and 2 articles were treated with LDXGT combined with western medicine compared with WM. In the study, the definition of western medicine was the conventional medicine used in clinical treatment of insomnia, including barbiturates, benzodiazepines, and nonbenzodiazepines. At present, benzodiazepines drugs are the first-choice drugs used for clinical treatment of insomnia, mainly including estazolam, alprazolam, diazepam, clonazepam, and lorazepam. Nonbenzodiazepines drugs were a new sedative hypnotic drug, with the main drugs including zolpidem and zopicron. Thus, 2 articles in the control group were treated with diazepam, 4 articles in the control group were treated with estazolam, 4 articles in the control group were treated with alprazolam, 2 articles in the control group were treated with routine treatment, and 1 article in the control group was treated with vitamin B1 and oryzanol. The clinical effective rate and cure rate were taken as evaluation indexes in all of the literature.

### 3.3 Methodological quality of the included trials

**Figure 2:**
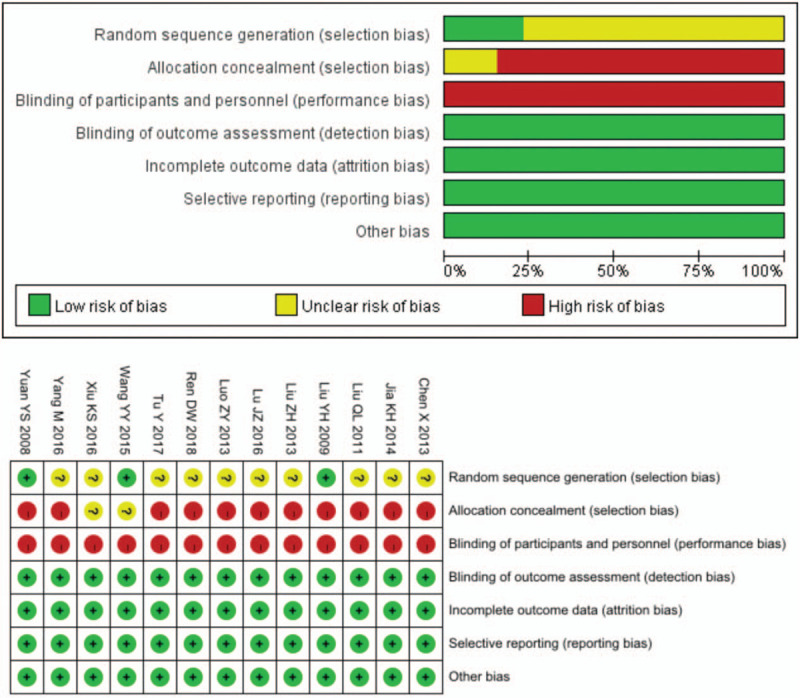
Graph of bias risk (a) and summary of bias risk (b). To review the authors’ judgement about each risk of bias for each included study; Graph of bias risk (a) and summary of bias risk (b).

Figure 2 summarizes the methodological quality of the 13 RCTs, and reviews the judgments about each risk of bias item for each included study. The methodological quality of the 13 included articles was generally low. Although all of them mentioned the use of random classification, they failed to mention the random numerical table method. None of these 13 studies made mention of the concealment of random distribution schemes, which may improve the therapeutic effectiveness. Blind method: all the 13 cases were blind, with only 2 being double blind. Lost-to follow-up: no cases of lost-to-follow-up. Others: there is no other bias, which is unclear about the risk of bias. In addition, there was no description of the clear blinding method, intentionality analysis, completeness of outcome data, or selectivity in reporting data results, which may also lead to the improvement to therapeutic effectiveness.

## 4 Meta analysis results

### 4.1 Effective rate analysis result

**Figure 3:**
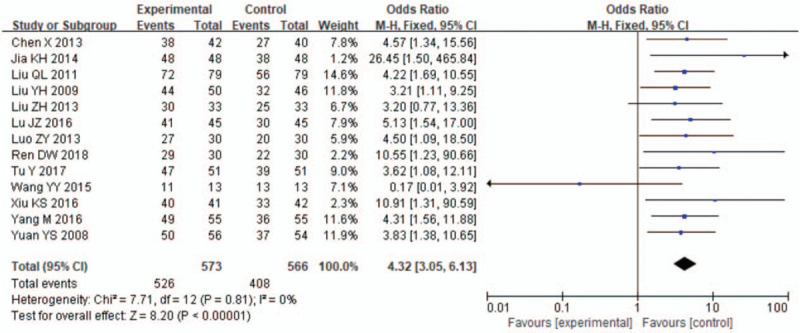
Forest plots of the clinical effective rate of LDXGT compared with western medicine.13 trials evaluated the effect of LDXGT in insomnia compared with the western drugs. The meta-analysis revealed a significant increasing effect of LDXGT.(total events:526 vs 406; 95% CI: 3.05 to 6.13; *I*^2^ = 0%, *P* < .00001); Forest plots of the clinical effective rate of LDXGT compared with western medicine.

The total effective rate was the most commonly used outcome measure to evaluate effectiveness. 13RCTs^[1–8,10–14,16]^ including 1181 patients provided data in the meta-analysis. Eleven meta-analysis reported the total clinical effective rate of LDXGT compared with western medicine, and the remaining 2 studies^[9,15]^ were compared with LDXGT and western medicine. Heterogeneity test shows that *P* = .81, including 2 groups which are comparable, *I*^2^ = 0%, OR = 4.32,95%CI [3.05,6.13]. The results of global effect test Z = 8.20 (*P* < .00001), and the Meta analysis showed that the total effective rate of LDXGT in the treatment of insomnia was higher than in the control group. (Fig. 3)

### 4.2 Cure rate analysis result

**Figure 4:**
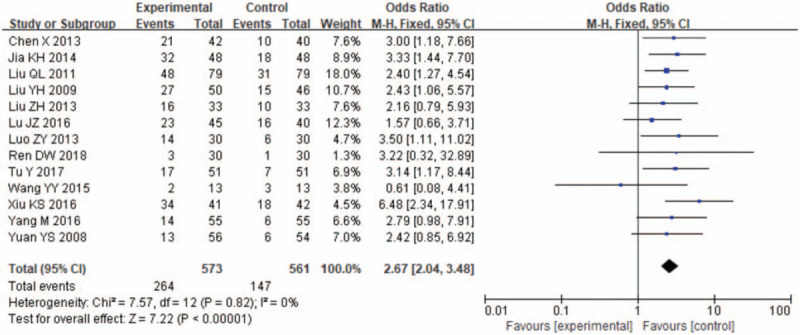
Forest plots of the clinical cure rate of LDXGT compared with western medicine. The cure rate was reported in all the 13 trials. Compared with LDXGT, cure rate was significantly lower in the control group.(total events:264 versus 147;95%CI:2.04 to 3.48; *I*^2^ = 0%, *P* < 0.00001); Forest plots of the clinical cure rate of LDXGT compared with western medicine.

The cure rate was taken as the evaluation index in all the 13 RCTs. This outcome measure was defined as cure of the signs and symptoms of insomnia in these trials, with no clinical heterogeneity found. Compared with conventional medicine treatment, LDXGT group significantly improved the cure rate. The results of heterogeneity test showed that *I*^2^ = 0%, for which the fixed-effect model was used. The results indicated that there was a significant difference in the clinical recovery rate of insomnia after post-treatment between LDXGT related therapy and western medicine alone. OR = 2.67, 95%CI [2.04, 3.48], Z = 7.22, *P* < .01, indicating that 2 groups of patients were statistically significant after treatment. (Fig. 4)

### 4.3 Publication bias

**Figure 5:**
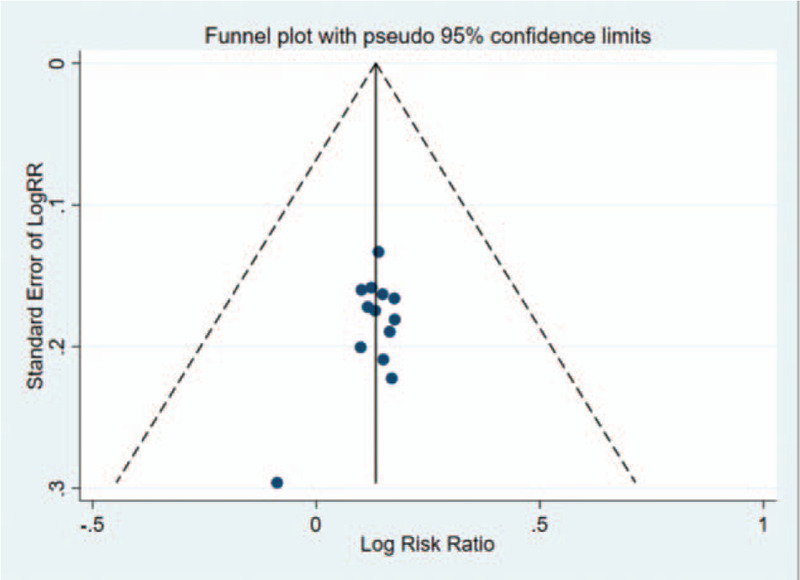
Funnel plot analysis of 13 trials for the outcomes were performed to explore the publication bias. The plot was symmetrical suggesting that the publication bias was not obvious; The total effective rate funnel plot of the experimental group and the control group.

**Figure 6:**
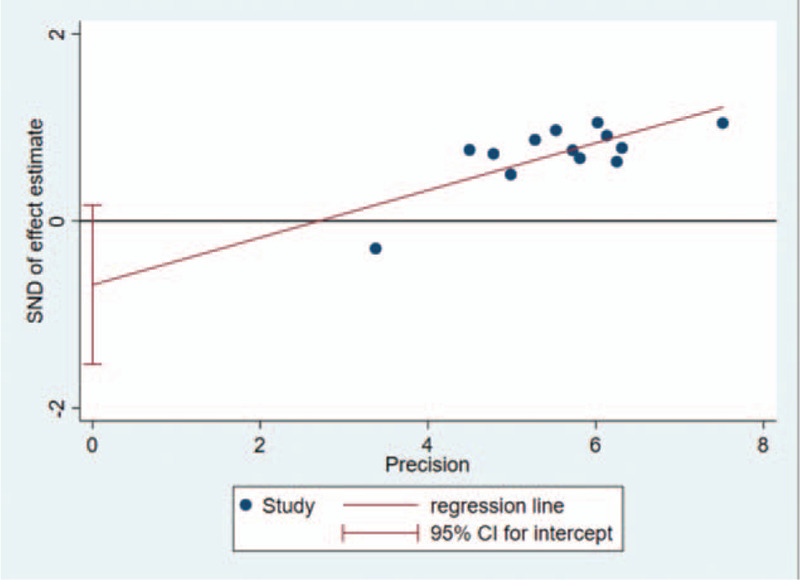
Egger test showed that there's no obvious publication bias in the literature.(slop=0.002;*P* > |t| = .106 > .05; 95%CI [–1.532296, 0.1699969]); Egger test of total effective rate between experimental group and control group; Egger test of total effective rate between experimental group and control group.

**Figure 7:**
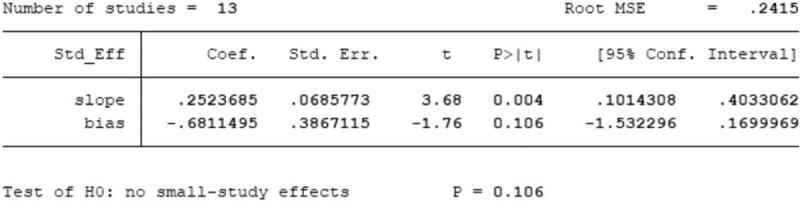
Egger test of total effective rate between experimental group and control group.

The shape of funnel plot could represent the Publication bias of the literature. In this study, the funnel plot demonstrated that it was basically symmetrical, suggesting that the bias is small and the conclusion is basically reliable (Figs. 5–7). The Egger test showed that: slop = 0.002, bias (*P* > |t| = .106 > 0.05), 95%CI [−1.532296, 0.1699969], including 0, which indicated that there is no obvious publication bias in the literature and confirmed the robustness of our results.

### 4.4 Adverse events

No significant adverse events were reported in any of the included trials. Adverse event monitoring was only reported in 1 study,^[13]^ whereas it was in the control group. Among the control group, there were 3 cases of dizziness, 3 cases of fatigue, 2 cases of dry mouth and 1 case of diarrhea. No adverse events were made mention of in the other studies.

## 5 Discussion

Longdan Xiegan decoction is a common prescription in traditional Chinese medicine, which has a positive clinical effect in the treatment of insomnia with hyperactivity of liver-fire, which shows a bad temper symptom. In this study, the clinical effectiveness and safety of LDXGT in the treatment of insomnia were systematically evaluated. The meta-analysis demonstrated that the total effective rate and cure rate of LDXGT in the treatment of insomnia were higher than that of the control group, and the results were statistically significant. The adverse reactions of LDXGT in the treatment of insomnia were less significant than those of western medicine, and the clinical safety of LDXGT was higher than that of western medicine group. Meanwhile, traditional Chinese medicine treatment of insomnia is highly beneficial to alleviate physical symptoms and reduce negative emotions. It can develop a healthy doctor–patient relationship and enhance mutual trust.

The limitation of this study is that most of the researches included in this system evaluation were not of high quality and lacked quality control. Besides, the literature in this study made no mention of the basis of sample size estimation. Although the method of random distribution was mentioned, the concealment of random distribution scheme was yet to be confirmed, and only 2 of all the studies mentioned the use of blind method. However, its implementation could not be determined. Therefore, to a certain extent, the external authenticity and credibility of the research results were reduced. The included studies lacked long-term follow-up data, which was of great significance to the better evaluation of LDXGT for the treatment of insomnia. The evaluation index selected by this study was subjective, and the influence of human factors on its authenticity may be more significant. In addition, the lack of retrieval of relevant foreign literature may lead to selective bias of the literature.

Although the quality of this paper is not high, it can exert influence on the results of Meta to some degrees. However, the conclusions show certain reference value for clinical application. We expect to conduct more high quality research in clinical trials of traditional Chinese medicine in the future, especially multicenter, large sample, random double blind controlled trials, so as to improve the overall quality of the literature, and obtain firm evidence-based medical evidence to validate the therapeutic effectiveness and safety of LDXGT in the treatment of insomnia.

## Author contributions

**Data curation:** Xiaomeng Sun.

**Formal analysis:** Yi Xu.

**Investigation:** Xiaoyu Zhang.

**Methodology:** Zhuang Su.

**Project administration:** Xu Fan.

**Resources:** Xiaomeng Sun, Yanying zhao.

**Software:** Shichao Nie, Jie Yang.

**Validation:** Dongyang Tan.

**Writing – original draft:** Sihang Xie, Liu Feng, Mingyi Gu.

**Writing – review & editing:** Xiaomeng Sun.

Xiaomeng Sun orcid: 0000-0001-7599-153X.
